# Why looking at the whole hippocampus is not enough—a critical role for anteroposterior axis, subfield and activation analyses to enhance predictive value of hippocampal changes for Alzheimer’s disease diagnosis

**DOI:** 10.3389/fncel.2014.00095

**Published:** 2014-03-31

**Authors:** Aleksandra Maruszak, Sandrine Thuret

**Affiliations:** Centre for the Cellular Basis of Behaviour, Department of Neuroscience, Institute of Psychiatry, King’s College LondonLondon, UK

**Keywords:** Alzheimer’s disease, hippocampus, hippocampal volume, hippocampal asymmetry, hippocampal shape, hyperactivation, dorsal hippocampus, ventral hippocampus

## Abstract

The hippocampus is one of the earliest affected brain regions in Alzheimer’s disease (AD) and its dysfunction is believed to underlie the core feature of the disease-memory impairment. Given that hippocampal volume is one of the best AD biomarkers, our review focuses on distinct subfields within the hippocampus, pinpointing regions that might enhance the predictive value of current diagnostic methods. Our review presents how changes in hippocampal volume, shape, symmetry and activation are reflected by cognitive impairment and how they are linked with neurogenesis alterations. Moreover, we revisit the functional differentiation along the anteroposterior longitudinal axis of the hippocampus and discuss its relevance for AD diagnosis. Finally, we indicate that apart from hippocampal subfield volumetry, the characteristic pattern of hippocampal hyperactivation associated with seizures and neurogenesis changes is another promising candidate for an early AD biomarker that could become also a target for early interventions.

Alzheimer’s disease (AD) is the most common type of dementia in people over the age of 65, with a lifetime risk of 10.5% (Sperling et al., 2011). In 2013, there have been 44.35 million people affected by dementia worldwide and their number will reach 75.62 million in 2030 (Alzheimer’s Disease International, 2013). AD encompasses pre-dementia and dementia stage. It is characterized by a long asymptomatic period that transforms into mild cognitive impairment (MCI) and later to dementia due to AD.

Individuals with MCI are affected by significant cognitive impairments which are not sufficient to meet AD criteria but are beyond normal healthy aging features. The amnestic subtype of MCI (aMCI) is characterized by a higher likelihood of progressing to dementia (Petersen et al., 2009). However, the annual conversion rate is 5–10% and the majority of MCI individuals even after 10 years will not progress to clinical AD, or some will even improve cognitively (Mitchell and Shiri-Feshki, 2009). Therefore, the possibility to predict who from the MCI group will eventually progress to AD would be invaluable for planning patients’ therapy, counselling and clinical trials. Therefore, given that the neurodegenerative process leading to dementia due to AD begins more than a decade before clinical diagnosis is made (Jack et al., 2013), there is a quest for reliable techniques and biomarkers enabling diagnosis at the preclinical stage to provide opportunity for therapeutic intervention. These methods need to be widely available and relatively inexpensive to find application in the diagnosis of the millions of affected individuals worldwide.

With an integrative perspective of molecular and cellular biology as well as neuroanatomy, our review focuses on the recent advancements in hippocampal analyses employing widely available magnetic resonance imaging (MRI) scanners, and describes how it might impact on improved early AD diagnosis.

We discuss why looking solely at the hippocampal volume is not sufficient to diagnose early stage AD. We present the latest data on the hippocampal changes in AD and suggest how they can be employed to diagnose AD earlier than we have been doing so far. To our knowledge, our review is the first one to provide a comprehensive summary of neuroimaging studies focused on hippocampal subfields in the context of AD. We highlight the subfields that might enhance the predictive value of current diagnostic techniques. Moreover, we present how changes in hippocampal volume, shape, symmetry and activation are reflected by cognitive impairment as well as neuropsychiatric symptoms, and how they are linked with neurogenesis alterations. We discuss how measuring these changes might be applied in AD diagnosis. In addition, we demonstrate why both hippocampal neurogenesis and functional differentiation along the anterior-posterior longitudinal axis of the hippocampus deserve more attention in future studies by presenting their relevance for presymptomatic AD diagnosis. Finally, we indicate that apart from hippocampal subfield volumetry, the characteristic pattern of hippocampal hyperactivation associated with seizures and neurogenesis changes is a very promising candidate for both an early AD biomarker and target for early pharmacological interventions.

The hippocampus is composed of interconnected subfields with distinctive histological characteristics and specialized functions, including four fields of the Cornu Ammonis (CA1-4), dentate gyrus (DG) and the subiculum. The nomenclature of hippocampal subfields and their anatomical definitions vary among different authors, however, there is even less agreement on the location of boundaries between hippocampal head, body and tail.

The hippocampus has two major interconnected roles. It is involved in consolidation of some forms of memory, learning and emotional processing. It encompasses also one of the brain niches where adult neurogenesis occurs, i.e., in subgranular zone of the DG. Neurogenesis is essential for memory, learning and mood (Deng et al., 2010; Eisch and Petrik, 2012; Zainuddin and Thuret, 2012) and there is extensive evidence showing that these processes are altered in AD (Lazarov and Marr, 2013; Maruszak et al., 2013). We discuss further the link between changes in the hippocampus and neurogenesis alterations.

## Structural changes in the hippocampus as one of AD hallmarks

In most cases, AD is diagnosed when the hippocampus is already considerably damaged. The hippocampus is one of the major targets of AD hallmarks: neurofibrillary tangles (NFT), amyloid plaques and neuronal loss. NFT are intraneuronal aggregates of hyperphosphorylated microtubule-associated protein, tau. Their number and spatiotemporal distribution correlates positively with cognitive decline and progression of AD (Braak et al., 1998). First, they affect the brain in the entorhinal/perirhinal cortex, thereby disrupting the origin of the perforant pathway projection to the hippocampus. Then NFT target hippocampal cornu Ammonis (CA) subfields, association cortex, and finally primary neocortex. In the hippocampus NFT target first CA1, then subiculum, later CA2 and CA3 (Braak and Braak, 1991; Braak et al., 1993). NFT are paralleled by neuronal and synapse loss, although it remains unclear whether NFT are causative for these changes or represent a neuroprotective response (Serrano-Pozo et al., 2011). The spatiotemporal spreading of NFT is closely reflected by the progression of brain atrophy, and both NFT and atrophy measures strongly correlate with cognitive decline (Bobinski et al., 2000; Jack et al., 2002; Johnson et al., 2012).

The extracellular amyloid plaques, which are composed of aggregated amyloid β (Aβ) peptides of 39–42 amino acids in length, display less predictable spatiotemporal distribution than NFT. They accumulate mainly in the isocortex and their presence does not correlate with dementia severity (Braak and Braak, 1991; Thal et al., 2002; Giannakopoulos et al., 2003).

The third characteristic feature of AD, the progressive cerebral atrophy, encompasses hippocampal atrophy and can be visualized by MRI. For the majority of AD patients (with the exclusion of those with hippocampal sparing and limbic-predominant AD) hippocampal atrophy is one of the earliest detectable symptoms of ongoing neurodegeneration, thus it has been incorporated in the new diagnostic criteria for AD (Mormino et al., 2009; Dubois et al., 2010; Lo et al., 2011; Jack et al., 2013). Hippocampal atrophy progresses nonlinearly, expressing features of a sigmoidal trajectory as it accelerates during transition from MCI to AD dementia (Sabuncu et al., 2011; Jack et al., 2013). It starts to diverge from normal rate of atrophy around 5.5 years before clinical diagnosis of dementia (Ridha et al., 2006). Already 3 years before diagnosis hippocampal volume is reduced by about 10%, whereas AD patients at the mild disease stage are characterized by the volume reduced by ~15–20% relative to controls (Johnson et al., 2012).

Hippocampal volume is strongly associated with memory recall performance in the elderly (Reitz et al., 2009). Likewise, in aMCI, MCI and AD hippocampal atrophy positively correlates with measures of cognitive decline, such as Mini-mental state examination (MMSE) or Alzheimer’s Disease Assessment Scale-Cognitive Subscale (ADAS-Cog; Pennanen et al., 2004; Jessen et al., 2006; Jak et al., 2007; Schuff et al., 2009; Sabuncu et al., 2011; Frankó and Joly, 2013). AD patients are characterized by higher average rate of hippocampal volume loss than healthy age-matched controls, whereas MCI patients have an intermediate level of volume loss between AD and control individuals (Schuff et al., 2009). Moreover, hippocampal atrophy in MCI converters is more pronounced compared to non-converters (Devanand et al., 2007; Kantarci et al., 2009; Risacher et al., 2009; Costafreda et al., 2011).

It has been proposed that surface-based methods enabling three-dimensional hippocampal shape analysis might serve as a better tool to localize early hippocampal atrophy. This technique estimates surface changes which enable localisation of regions of highest inward and outward transformations. Hippocampal shape is predictive for dementia in the preclinical period, independently of age and gender (Achterberg et al., 2013). Moreover, combining both shape and volume enables better prediction of the risk of dementia (Achterberg et al., 2013). However, the surface-based methods have also some important drawbacks which will be discussed further in this review.

### Factors affecting hippocampal volume in AD

Hippocampus is a dynamic structure and there are several factors that influence its volume. First of all, hippocampal volume has been shown to be highly heritable as a recent meta-analysis of genome-wide association studies revealed (Stein et al., 2012). Moreover, there were also suggestions of ApoE epsilon4 (*APOE4*) effect on hippocampal volume. Together with some other AD risk factors, such as elevated homocysteine level, *APOE4* was associated with smaller hippocampal size and changes in hippocampal asymmetry (described below). For instance, Schuff et al. reported that the volume loss in AD patients was significantly higher if they were carriers of *APOE4* allele compared to *APOE4* non-carriers, irrespective of their cognitive impairments (Schuff et al., 2009). *APOE4* status was not associated with higher hippocampal volume loss in healthy controls or MCI patients (Schuff et al., 2009). Mueller and Weiner (2009) have shown that both AD patients and controls that were carriers of *APOE4* exhibit hippocampal volume loss specifically in CA3 and DG compared to non-carriers (Mueller and Weiner, 2009). Despite the long list of the *APOE4* roles in functional and gray matter changes throughout adulthood as it has been shown to play an important role in neuronal development, neuron migration, axon guidance, microtubule stability, dendritic spine density, synaptic plasticity, and regeneration after injury and in adult neurogenesis (Yang et al., 2011; Dean et al., 2014), there are also studies that do not find positive association of *APOE4* allele with hippocampal volume changes (Reitz et al., 2009; Wang et al., 2012) or that *APOE4* influences not the hippocampal volume but the rate of volume loss over time (Moffat et al., 2000; Cohen et al., 2001; Jak et al., 2007; Lyall et al., 2013). The discrepancies may stem from methodological variability between the studies, such as for instance different sample sizes or different approaches to comparing APOE genotypes.

Among other factors affecting hippocampal volume is age, thus hippocampus is independently affected both by age and AD pathology (Raz et al., 2005; Raji et al., 2009). In addition, many age-related pathological conditions, of which many increase AD risk, such as e.g., hypertension, diabetes mellitus, cardiovascular disease and head trauma, influence hippocampal volume (Fotuhi et al., 2012). Moreover, hippocampal volume might be affected by medications. For instance, antidepressants or cholesterol-lowering treatment have been shown to help reverse hippocampal atrophy (Fotuhi et al., 2012).

Among the factors increasing hippocampal volume is cognitive stimulation, mindfulness and aerobic exercise (for review see Fotuhi et al., 2012). The latter one has been linked with increased both right and left hippocampal volume by around 2% over 1 year and reversing age-related hippocampal shrinkage as well as improving cognition (Erickson et al., 2011). In a 9-year long prospective cohort study, it has been shown that regular physical activity, as simple as walking, increases hippocampal volume, and in addition, larger hippocampal volume was associated with decreased risk of cognitive impairment (Erickson et al., 2010). The influence of aerobic exercise on hippocampal volume might be of particular interest of individuals at risk of AD, as it seems to selectively increase the volume of the anterior hippocampus, which is affected early on in AD course (Erickson et al., 2011). Moreover, there is also extensive evidence of the role of exercise in increasing hippocampal neurogenesis as it increases proliferation and survival of hippocampal progenitor cells (Cotman and Berchtold, 2002; van Praag et al., 2005; Creer et al., 2010). Notably, the remaining above-mentioned factors influencing hippocampal volume have been also implicated in neurogenesis. Recently, another common factor between neurogenesis and hippocampal volume has been revealed—brain-derived neurotrophic factor, which level is decreased in AD as well as in impaired neurogenesis (Honea et al., 2013).

### Hippocampal asymmetry in AD

Although hippocampus is structurally and functionally asymmetric, right (R) vs. left (L) hippocampal volume differences have received less research attention. In healthy adults there is hemispheric asymmetry of the whole hippocampus, with larger volume of the right one (Pedraza et al., 2004; Shi et al., 2009). There are also right vs. left differences in the layers’ thickness and volumes of different hippocampal subfields. For instance, Lister et al. identified asymmetries in neuronal numbers in rat CA1 and CA3/CA2 subfields, with the right hemisphere containing 21 and 6% fewer neurons, respectively (Lister et al., 2006).

Hippocampal volume asymmetry has been connected with cognitive functions and it has been suggested that hippocampal subfields analysis should be included in these correlation studies. For instance, Woolard and Heckers (2012) in a study of 110 healthy individuals of 32.3 ± 10.7 years of age demonstrated that the R > L asymmetry is limited to the anterior hippocampus and it is correlated with a measure of general cognitive functions (Screen for Cognitive Impairment in Psychiatry) (Woolard and Heckers, 2012). Moreover, they showed that the volume of anterior hippocampus correlates with the volumes of all four cortical lobes, whereas the posterior hippocampus volume was found strongly correlated with the volume of occipital cortex (Woolard and Heckers, 2012).

A meta-analysis indicated that in AD there is a significant R > L hippocampal volume asymmetry as compared to the control group (Barnes et al., 2005; Shi et al., 2009). Given that hippocampal asymmetry might be due to genetic, developmental and environmental factors (Verstynen et al., 2001; Tang et al., 2008), hippocampal asymmetry in AD has been suggested to be influenced by the dose effect of the *APOE4* allele; the R > L asymmetry is progressively reduced and even reversed in *APOE4/4* carriers affected by AD (Geroldi et al., 2000). It has been also postulated that the left hippocampus is more vulnerable than the right one to AD pathology due to already smaller volume (Muller et al., 2005). Nevertheless, hippocampal asymmetry changes with AD progression, with the left hippocampus affected first by dementia, followed by atrophy in the right hippocampus after a time lag (Thompson et al., 2004; Morra et al., 2009b). Finally, one needs to be mindful of the limitations associated with using manual asymmetry measurements. Recently, Maltbie et al. performed a semi-automatic hippocampal segmentation and pointed out that neuroimaging is typically biased to one side due to laterality in visual perception (Maltbie et al., 2012). Although this bias has been reported to be smaller than the true anatomical R > L hippocampal asymmetry values (for further information on this topic and hints on avoiding the potential bias, please refer to work by Rogers et al., 2012), it is worth to take it into consideration while interpreting hippocampal asymmetry data.

### The supremacy of subfield analysis over total hippocampal volume measurements for AD diagnosis

Similarly to other AD biomarkers, application of hippocampal volume changes for AD diagnosis has some limitations. Although there is a higher rate of global hippocampal volume loss in AD patients compared to control individuals and MCI patients, changes in hippocampal volume show moderate sensitivity and low specificity to AD as it is observed also in other conditions such as semantic dementia (Frisoni et al., 2010; Lindberg et al., 2012; Tondelli et al., 2012; La Joie et al., 2013). Sensitivity of total volume analysis is restricted to the stages from MCI to moderate dementia stage (Frisoni et al., 2010). In the asymptomatic AD the markers of Aβ deposition (carbon 11-labeled Pittsburgh Compound B (PiB) positron emission tomography (PET), cerebrospinal fluid (CSF) Aβ_42_) have been suggested to outperform markers of structural changes but their values plateau by MCI stage (Frisoni et al., 2010).

Given that the hippocampal subfields are differentially vulnerable to neuropathology in AD and their measurements have been shown to be more accurate than global hippocampal volumetry to differentiate prodromal AD (aMCI) from cognitively normal controls (La Joie et al., 2013), it has been proposed that hippocampal subfield volumetry might be a better biomarker for early detection of AD.

AD is characterized by the most prominent neuron loss in CA1 which correlates with dementia severity and Braak staging (Rossler et al., 2002; Mueller et al., 2010). Other studies indicated that apart from smaller volume of CA1, AD patients have decreased size of entorhinal cortex (EC), subiculum and CA1-2 transition zone (Mueller and Weiner, 2009; Mueller et al., 2010; Apostolova et al., 2012) and these findings are consistent with the level of neuronal loss (West et al., 2004; Zarow et al., 2005).

Likewise, owing to differences between studies such as age of participants, applied segmentation protocols, MRI resolution, limited statistical power and small effect sizes, there are still controversies as to which hippocampal subfield is the most reliable to distinguish normal healthy controls from aMCI. For instance, CA1 is the earliest affected hippocampal subfield by NFT and neuronal loss (Braak and Braak, 1991; Braak et al., 1993; West et al., 1994) and it has been shown to remain relatively preserved in healthy aging, thus it has been proposed that CA1 atrophy might be a biomarker of presymptomatic AD (Csernansky et al., 2005; Scher et al., 2007; Frisoni et al., 2008; Gerardin et al., 2009; Raji et al., 2009; Apostolova et al., 2010; La Joie et al., 2010). However, there are other candidates for the area of the largest difference between aMCI and controls, as some indeed point to CA1 (Yassa et al., 2010; Pluta et al., 2012; La Joie et al., 2013), however, others suggest that it is CA1-2 transition area (Mueller and Weiner, 2009; Mueller et al., 2010) but subiculum and CA2-3 (Hanseeuw et al., 2011) or CA3/DG (Yassa et al., 2010) have been also proposed. Some researchers did not focus on aMCI but on the whole MCI instead. Apostolova et al. (2006) showed that MCI patients with smaller hippocampi, particularly in the CA1 and subiculum, are at a higher risk of converting to AD (Apostolova et al., 2006). Moreover, MCI individuals that improve cognitively and revert to control status are characterized by larger hippocampal volumes and relative sparing of CA1 and subiculum (Apostolova et al., 2006). In addition, in a later study Apostolova et al. reported that the most pronounced differences between MCI and AD were in CA1 and CA2-3 bilaterally (Apostolova et al., 2012). Although there is no general agreement about the potential role of CA1 in AD diagnosis, it has been shown that the CSF tau and Aβ_42_ concentrations at baseline correlate with the rate of hippocampal atrophy and progressive inward deformations of the CA1 subfield in the individuals at the very mild stage of AD (Clinical Dementia Rating 0.5; Wang et al., 2012).

In general, the problem with modelling hippocampal volume or hippocampal subfields volume changes in AD is that neuronal loss is detected only in some AD mouse models. Some tg-mice exhibit CA1 pyramidal neuron loss (i.e., Wright et al., 2013; Beauquis et al., 2014), however there are also reports of no change in number of neurons (Schaeffer et al., 2011). For instance, in PDAPP mice at 5 months of age (before the appearance of amyloid plaques in the hippocampus and cortex), a 12% decrease in CA1 and 25% reduction in DG volume was detected (Beauquis et al., 2014). Smaller DG size in PDAPP mice has been reported also by their researchers (Redwine et al., 2003; Wu et al., 2004). Atrophy of CA3/DG has been found in aMCI patients (Yassa et al., 2010; Atienza et al., 2011). However, in MCI and AD patients there have been no reports of sole DG atrophy. Mueller et al. (2010) argue that it might be due to the fact that DG and CA3 are frequently analysed together and owing to the relatively good preservation of CA3 in AD, it is possible that it overshadows subtle effects in DG (Mueller et al., 2010). Indeed, although surface-based methods have similar prognostic performance to other structural neuroimaging approaches, these methods do not provide quantitative information about hippocampal subfields volumes. Moreover, as Pluta et al. argue, that automatic hippocampal subfield segmentation basing on surface-based parcellation does not adequately model the CA4/DG subfield, given that it is to a substantial extent internal to the hippocampal formation (Pluta et al., 2012). Therefore, changes in the hippocampal surface might not truly reflect degeneration in DG.

### Anterior vs. posterior location of hippocampal subfields and their role in AD

Another approach to looking at the hippocampus employs differences in gene expression, behavior and functional connectivity, which were used to divide rodent hippocampus into three gross anatomical subregions, following the longitudinal axis of the hippocampus: dorsal, ventral and intermediate subfields (see Poppenk et al., 2013 for detailed information about connectivity and detailed information about postulated differences along dorsal-ventral axis; Moser and Moser, 1998; Fanselow and Dong, 2010; Zarei et al., 2013). These hippocampal regions correspond to the septal, temporal and caudal poles in rat (Swanson and Cowan, 1977). The septal pole, located dorsally and anteriorly in rodents, corresponds to posterior hippocampus in humans (Tanti and Belzung, 2013). The temporal pole, ventrally and posteriorly located in rodents, is the anterior hippocampus in humans (Tanti and Belzung, 2013). The intermediate subregion in humans is often not distinguished (Poppenk et al., 2013).

These three hippocampal regions are characterized by different patterns of connectivity with prefrontal cortex (PFC), posterior cingulate cortex (PCC) and thalamus (Zarei et al., 2013). For instance, connections between the hippocampus and PCC are thought to be involved in autobiographical and episodic memory (Zarei et al., 2013).

So far, there have been strong discrepancies in defining the boundaries of the dorsal/ventral subregions (see review by Tanti and Belzung, 2013). Frequently, little information regarding the methodology behind distinguishing the dorsal and ventral subfields is provided which prevents comparisons between studies. Nevertheless, the major hippocampal subfields, such as CA1, CA3 and DG are segregated into dorsal, intermediate and ventral.

The most pronounced atrophy in AD has been found in the anterior hippocampus (as well as posterior parahippocampal gyrus and the precuneus) compared to age-matched controls (Raji et al., 2009). Moreover, hippocampal head (anterior) atrophy has been reported as a predictive marker of conversion to AD (Csernansky et al., 2005; Apostolova et al., 2006; Morra et al., 2009a; Costafreda et al., 2011). Costafreda et al. identified more pronounced changes in the right lateral and medial aspects of hippocampal head in individuals that later converted to AD and his results correspond with previous reports of anterior CA1 (Csernansky et al., 2005; Apostolova et al., 2006; Costafreda et al., 2011). Recently, Franko and Joly demonstrated that regions with the highest atrophy rate in anterio-lateral hippocampus correspond to those with highest tau deposition (Frankó and Joly, 2013). This supports the above-mentioned link between anterior CA1 and conversion to AD.

In addition, AD patients exhibit weaker connectivity with the PCC in the hippocampal body and stronger connectivity with the PFC in the head of the hippocampus compared to the healthy controls (Zhang et al., 2010; Zarei et al., 2013). Previous studies found disrupted functional connectivity with default mode network (DMN) in AD (as it is not in the scope of this review, please refer to Greicius et al., 2004; Mevel et al., 2011). Moreover, diffusion tensor imaging (DTI) provided evidence that structural connectivity between PCC and hippocampus is decreased during the earlier stages of AD (Zhou et al., 2008). The differences in the hippocampal connectivity were proposed to contribute to cognitive deficits in AD (Zarei et al., 2013).

Interestingly, the levels of adult neurogenesis in the dentate gyrus of the hippocampus have been associated with differential functional roles dependant on its dorsal (posterior) or ventral (anterior) location: dorsal neurogenic pool of hippocampal progenitor cells is associated with learning and memory whereas ventral—with motivational and emotional behaviors. The latter one can be exemplified by the studies looking at neurogenesis in neuropsychiatric disorders, such as depression, in which greater vulnerability to stress is reflected by a significant decrease in the ventral hippocampus (Tanti and Belzung, 2013). Given that neurogenesis is altered in the course of AD (for details see review by Lazarov and Marr, 2013), and that dorsal neurogenesis is implicated in memory and learning, it is surprising that only few studies have explored the role of dorsal-ventral differences in AD and in hippocampal neurogenesis in the course of AD.

Fuster-Matanzo et al. identified that overexpression of glycogen synthase kinase-3β (GSK-3β), an enzyme involved in both AD pathogenesis and neurodevelopment, leads to significant decrease in the number of granular neurons and increased astrocytosis in mice DG (Fuster-Matanzo et al., 2011). Notably, these changes are spared in the ventral DG, where reduced GSK-3β activity and less cell death compared with the dorsal area were found (Fuster-Matanzo et al., 2011). Fuster-Matanzo et al. study has elegantly demonstrated that there are regional differences in GSK-3β activity in AD which might explain why dorsal hippocampus is more susceptible to neurodegeneration (Fuster-Matanzo et al., 2011).

In the 3xTg mice the level of proliferation was reduced compared to non-Tg animals, and in the female mice it was present predominantly in the dorsal than ventral hippocampus, whereas the males exhibited first changes in neurogenesis in the ventral hippocampus (Rodriguez et al., 2008). However, the authors did not investigate if the number of new-born neurons is subregion-dependent.

In another report, authors did not analyse neurogenesis but presented interesting findings with regard to dorsal hippocampus and spatial memory, for which the dorsal hippocampus is essential. Yiu et al. found that TgCRND8 mice exhibit severe impairment of spatial memory and presented with decreased CREB activation in dorsal CA1, decreased spine density and dendritic complexity of CA1 pyramidal neurons (Yiu et al., 2011). They exhibited also decreased neuronal network activity.

These three studies point to the role of dorsal hippocampus in AD. However, as mentioned already above, the anterior (ventral in rodents) hippocampus, is the subregion, where the majority of volume differences are reported in aMCI and AD patients. Wolf et al. reported that atrophy of left posterior hippocampus was better in discriminating controls from MCI (Wolf et al., 2001). It is plausible that neurogenesis impairment in AD patients affects predominantly mood, whereas circuits’ dysfunction underlies memory impairment and associated symptoms. Hence, the described above dorsal-ventral (posterior-anterior) discrepancies in the hippocampus role in the early stages of AD might be reflected by neuropsychiatric symptoms which receive much less attention than cognitive symptoms, but constitute a common feature of AD (Lyketsos et al., 2011). Around 35–75% MCI (Apostolova and Cummings, 2008) and 75% of AD patients experience emotional symptoms, with depression and anxiety as the most common ones during the prodromal disease stage (Sturm et al., 2013). The most frequently observed neuropsychiatric features in MCI individuals are apathy, depression, anxiety, irritability, whereas in AD patients apart from these same symptoms agitation/aggression is also present (Lyketsos et al., 2002; Wadsworth et al., 2012; Dillon et al., 2013). It has been noticed that these symptoms often precede and accelerate conversion to dementia (Apostolova and Cummings, 2008; Wadsworth et al., 2012; Dillon et al., 2013). For instance, depression has been shown to double risk of conversion to AD (Modrego and Ferrández, 2004). Notably, emotional symptoms accompanying cognitive problems are more common among future converters to AD (Gallagher et al., 2011). However, neuropsychiatric symptoms do not correlate with cognitive impairment (Dillon et al., 2013). Notably, the data from AD animal models frequently does not cover emotional symptoms, thus dorsal-ventral analysis might by biased by focusing exclusively on the cognitive domain.

Another emotion-related AD feature is emotion contagion which is a basic affective mechanism synchronizing physiological and behavioral states with those of another to promote affective simulation and altruistic behavior and is not dependant on higher order cognitive processes. Anterior hippocampus has been associated in both MCI and AD patients with experiencing high levels of emotional contagion (Sturm et al., 2013). Emotional contagion was found to be weakly correlated with depression symptoms (Sturm et al., 2013). It has been proposed that higher level of emotional contagion might be due to less efficient inhibition of emotions and salience network release which are associated with smaller volume in primarily right-hemisphere temporal lobe structures (Sturm et al., 2013).

## Functional hippocampal abnormalities in AD

In the next section of the review, we focus on the link between functional hippocampal changes and their predictive value for AD, and we demonstrate the link between structural and functional alterations is still unresolved. These changes could occur sequentially or simultaneously and they might influence each other, as we exemplify by showing the association between neurogenesis and seizures.

### Hyperexcitability and seizures at early AD stages

It has been reported that during memory tasks individuals with mild AD show reduced hippocampal activity, whereas aMCI and MCI patients exhibit hyperactivity in the hippocampus/parahippocampal region (Hämäläinen et al., 2007; Miller et al., 2008; O’Brien et al., 2010; Yassa et al., 2010; Putcha et al., 2011). Task-related hyperactivity has been described in asymptomatic carriers of AD pathological mutations during associative encoding task (right anterior hippocampus; Quiroz et al., 2010); in asymptomatic offspring of autopsy-confirmed AD patients (Bassett et al., 2006); in cognitively intact young and old carriers of *APOE4* (Bookheimer et al., 2000; Dickerson et al., 2005; Filippini et al., 2009) and in low-performing clinically healthy aged individuals (Miller et al., 2008). Conversely, individuals at late aMCI stage and early AD already express the hippocampal hypoactivity pattern (Hämäläinen et al., 2007). During an associative memory task hyperactivation of the anterior hippocampus and EC in MCI patients compared to the controls was observed (Dickerson et al., 2005). Recently, the characteristic pattern of hyperactivity as well as shape and volume changes were detected in the CA3/DG of aMCI patients (Yassa et al., 2010; Bakker et al., 2012). CA3/DG network is essential for a key process for episodic memory, pattern separation (described later in this review).

Initially this type of hyperactivity has been regarded as compensation to deal with a cognitive task by recruiting additional neuronal resources; however it is more likely that it reveals neuronal excitotoxicity and in addition, the excess activation directly contributes to memory impairment (Brewer et al., 1998; Morcom et al., 2007; Putcha et al., 2011). There is an overall negative correlation between hippocampal activity and performance on memory task in the aged individuals (Putcha et al., 2011). Therefore, given that the network hyperexcitability contributes to cognitive impairment, Bakker et al. have demonstrated that by using a low dose of antiepileptic drug, levetiracetam, hippocampal activation in aMCI was reduced to the level observed in the control group and that had an impact on improved memory performance (Bakker et al., 2012).

In AD patients as well as in several transgenic AD mouse models spontaneous epileptic seizures have been observed (Velez-Pardo et al., 2004; Rao et al., 2009; Noebels, 2011) and they are attributed to the above-described increased network hyperexcitability (Hazra et al., 2013). Hazra et al. suggested that the seizures occur due to failure of inhibitory interneurons in DG to generate action potentials which leads to impaired DG function that is analogous to that observed in epilepsy. In addition, there is increased synaptic facilitation in the perforant pathway, leading to increased excitatory synaptic responses and spatiotemporal hyperexcitability of the DG perforant pathway circuit, and as a consequence a runaway excitation affecting hippocampal circuits adjacent to DG, which is called “breakdown of DG” (Hsu, 2007; Busche et al., 2008, 2012; Noebels, 2011; Hazra et al., 2013). These phenomena have been attributed to the Aβ pathology that has been linked with synaptic depression and to LTP inhibition (Busche et al., 2008; Hazra et al., 2013).

The occurrence of seizures in AD might have serious implications for neurogenesis. Seizures have been shown to increase neurogenesis, however they eventually decrease the survival of new-born neurons which also show aberrant migration and form aberrant circuits (see review by Parent and Murphy, 2008). Recently, Hester and Danzer (2013) demonstrated that status epilepticus similarly affects both dorsal and ventral hippocampus integration of new-born granule neurons and mossy fiber sprouting (Hester and Danzer, 2013).

Seizures are followed by comorbidities such as emotional difficulties (including increased risk for depression), cognitive impairment (memory problems) and behavioral problems, and at least some of them originate in disrupted neurogenesis. Surprisingly, there is considerable overlap between these symptoms and AD at an early stage.

### Pattern separation in AD

Hyperactivity in DG/CA3 region has been linked with age-related mnemonic discrimination deficits, i.e., an inability to distinguish between items and similar objects—so called lures in memory (Yassa et al., 2011a). Aging is characterized by a shift in bias from pattern separation to pattern completion in the DG/CA3 network and that correlates with changes in perforant pathway integrity (Yassa et al., 2011b). Perforant pathway originates in the layer II of EC and leads to granule cells in the DG which send projections to CA3. That is an essential network for pattern separation—encoding distinctive representations of experiences that have overlapping features with prior memories (Gallagher and Koh, 2011). DG plays an essential role in pattern separation, whereas CA3 in pattern completion (Yassa and Stark, 2011).

As it has been already mentioned above, AD is characterized by a degeneration of perforant pathway connections and compromised pattern separation circuit might lead to episodic memory impairment. Recently, Ally et al. reported that AD patients demonstrate impaired both pattern separation and completion, whereas individuals with aMCI, depending on the lag between tasks, behaved similarly to the controls (if the lag was short) or to AD patients (if the lag was long) which indicates a rapidly degrading visual memory representation in aMCI (Ally et al., 2013). Therefore, the authors proposed that aMCI is characterized by rapid forgetting, whereas dementia due to AD is a disorder of encoding. Moreover, in 12-month old Tg2576 AD mice expression of mutant human APP led to disruption of DG/CA3 network, which is proposed as critical for pattern separation (Palmer and Good, 2011).

There is also accumulating data showing that hippocampal neurogenesis is an important player for pattern separation (Aimone et al., 2010, 2011; Sahay et al., 2011a, b; Tronel et al., 2012). Therefore, it is plausible that neurogenesis alterations in the course of AD are contributing to impairment of pattern separation, however, it remains to be investigated.

Given that the volume of the posterior hippocampus positively correlates with memory for very detailed contextual information (temporal and spatial relations; representation at fine granularities), whereas anterior hippocampus is responsible for a coarse, global representation (spatial locales or contexts, coarse representation), it has been proposed that posterior hippocampus is biased towards pattern separation, whereas anterior hippocampus is more suited for pattern completion (Poppenk and Moscovitch, 2011; Evensmoen et al., 2013; Poppenk et al., 2013). Moreover, Poppenk and Moscovitch (2011) suggested that the posterior, but not the anterior hippocampus, is closely linked to episodic memory retrieval through its connection with the DMN, including pregenual anterior and PCC, and precuneus (Poppenk and Moscovitch, 2011). It remains to be explored how the reported decrease in the anterior hippocampal volume observed in AD corresponds with these hypotheses.

## Conclusions

There is considerable evidence that hippocampal volume decreases early during AD progression. However, these symptoms are not specific enough to fulfil criteria of AD biomarkers and enable presympromatic AD diagnosis. In this review, we present current data on detailed subfield and subregion analyses of the hippocampus and we demonstrate that so far, there is no consensus on which subfield could become a reliable early-stage disease biomarker. There are discrepancies in the analysed cohorts, methodology (labor-intensive and suffering from intra- and interobserver variability manual segmentation vs. automatic segmentation protocols; using T1-weighted MRI sequences at 1.5–3 Tesla that lack the contrast and resolution to distinguish e.g., CA4/DG region vs. T2-weighted MRI), using single or multiple MRI scans over time, anatomical labelling (e.g., in delineating the border between the anterior and posterior hipocampus), and that all prevents direct comparisons between studies. For instance, Nestor et al. argue that absolute volume differences between different protocols may vary by > 30% (Nestor et al., 2012). Our review indicates that apart from hippocampal subfield volumetry, there is another promising candidate for early AD biomarker which is the characteristic pattern of hippocampal hyperactivation associated with seizures and neurogenesis changes. The relation between these phenomena demands also further in depth investigations as it might unravel a very important early disease target for therapeutic intervention. Finally, we believe that improving harmonization of different MRI protocols and in depth analysis of neurogenesis changes in the course of AD might result in development of new disease cellular and molecular signatures.

## Conflict of interest statement

The authors declare that the research was conducted in the absence of any commercial or financial relationships that could be construed as a potential conflict of interest.
